# An integrated *in-silico* approach for drug target identification in human pathogen *Shigella dysenteriae*

**DOI:** 10.1371/journal.pone.0303048

**Published:** 2024-05-16

**Authors:** Hurria Qureshi, Amina Basheer, Wasim Sajjad, Muhammad Faheem, Syed Babar Jamal

**Affiliations:** 1 Department of Biological Sciences, National University of Medical Sciences, Islamabad, Pakistan; 2 Department of Biomedical Sciences, University of North Dakota, School of Medicine and Health Sciences, Grand Forks, ND, United States of America; Hawassa University College of Medicine and Health Sciences, ETHIOPIA

## Abstract

*Shigella dysenteriae*, is a Gram-negative bacterium that emerged as the second most significant cause of bacillary dysentery. Antibiotic treatment is vital in lowering *Shigella* infection rates, yet the growing global resistance to broad-spectrum antibiotics poses a significant challenge. The persistent multidrug resistance of *S*. *dysenteriae* complicates its management and control. Hence, there is an urgent requirement to discover novel therapeutic targets and potent medications to prevent and treat this disease. Therefore, the integration of bioinformatics methods such as subtractive and comparative analysis provides a pathway to compute the pan-genome of *S*. *dysenteriae*. In our study, we analysed a dataset comprising 27 whole genomes. The *S*. *dysenteriae* strain SD197 was used as the reference for determining the core genome. Initially, our focus was directed towards the identification of the proteome of the core genome. Moreover, several filters were applied to the core genome, including assessments for non-host homology, protein essentiality, and virulence, in order to prioritize potential drug targets. Among these targets were Integration host factor subunit alpha and Tyrosine recombinase XerC. Furthermore, four drug-like compounds showing potential inhibitory effects against both target proteins were identified. Subsequently, molecular docking analysis was conducted involving these targets and the compounds. This initial study provides the list of novel targets against *S*. *dysenteriae*. Conclusively, future *in vitro* investigations could validate our *in-silico* findings and uncover potential therapeutic drugs for combating bacillary dysentery infection.

## Introduction

*S*. *dysenteriae* bacterium belongs to rod-shaped family Enterobacteriaceae, which is the main cause of dysentery [1]. It has been reported that around 200,000 yearly mortalities from diarrhoea are attributed to shigellosis, a serious public health hazard around the world, with a significant proportion of deaths occurring among children below the age of five [2]. Shigellosis is characterized by sporadic outbreaks in developed countries, whereas in developing nations, reported cases are likely to be approximately 20 times higher than in developed countries. Nevertheless, most of the cases remain unreported in these regions. It is expected that if no action is taken, this number is expected to increase to 4.2 million in Africa and 10 million worldwide by 2050 [3].

There are numerous instances of transmission occurring through faeces-contaminated food and water, but other recognised methods of infection include flies and fomites. *Shigella* is an extremely virulent human pathogen, and one of its remarkable characteristics is the minimal number of organisms required to cause illness approximately infectious threshold can be as few as 100 organisms [4]. *S*. *dysenteriae* causes illness by invading and damaging the epithelial cells that line the colon, ileum, and rectum in the gastrointestinal tract. This invasion disrupts the normal functioning of these cells, leading to symptoms such as diarrhoea, abdominal cramps, and fever [5].

The use of antibiotics to treat enteric bacterial infections is crucial for minimizing disease prevalence and fatality rates. The misuse or overuse of antibiotics in the treatment of diarrhoea contributes to the escalation of antibiotic resistance. Multi-drug resistance is the major problem observed in *S*. *dysenteriae* worldwide [6]. There are several mechanisms in *S*. *dysenteriae* through which *Shigella* may end up resistant to the antibiotics such as expel of drugs by efflux pumps, less penetration in cell and increased modification of drugs and deactivating the enzymes [6].

Due to the growing prevalence of resistance to ciprofloxacin and ceftriaxone, azithromycin is considered a final option, United States Food and Drug Administration (FDA)-approved antimicrobial agents, particularly for managing systemic infections, including those attributable to *Shigella* spp [7]. The current study is designed to utilize a range of computational tools and techniques to identify a potential drug target for combating infectious diseases caused by *S*. *dysenteriae*. We have identified two potential virulent protein targets of *S*. *dysenteriae* that are Integration host factor subunit alpha and Tyrosine recombinase XerC. The Integration host factor alpha subunit is one of the subunit proteins which is a heterodimeric protein, involve in the genetic recombination. In *Shigella*, it plays a crucial role in the regulation of the expression of multiple virulence genes [8]. The XerC protein, which is highly conserved, plays a crucial role in resolving chromosome multimers by DNA replication in prokaryotic organisms [9]. XerC recombinases belong to the superfamily of tyrosine site-specific recombinases, which constitutes a vast group of enzymes responsible for catalysing DNA cleavage and re-joining by employing a conserved tyrosine nucleophile. Tyrosine recombinases facilitate a variety of programmed DNA rearrangements, including the mobilization of virulence factors [10]. These two proteins exhibited promise as potential drug targets based on their virulence.

In this study, plant-derived compounds were employed as antibacterial agents against prioritized target proteins. We used an extensive range of bioinformatics techniques, including pan-genomics, subtractive analysis, and comparative analysis, to identify *S*. *dysenteriae* strain SD197 genomic data and its proteome This approach helped to refine the selection of lead compounds for addressing *S*. *dysenteriae* infections. A pan-genome approach was utilized to compare the proteomes of the 27 genomes of *S*. *dysenteriae*, and only genes that were found in each strain of *S*. *dysenteriae* were chosen for further analysis.

## Materials and methods

### Collection of genomes and proteome

In this study, a comprehensive pan-genome analysis was conducted, encompassing the complete genome sequences of all 27 strains of *S*. *dysenteriae*. The reference genome and proteome sequences for these strains were retrieved from National Centre for Biotechnology Information (NCBI) and UniProt, respectively [11].

### Identification of core genomes

The core genome of *S*. *dysenteriae* was identified employing the Efficient Database framework for comparative Genome Analyses using BLAST score Ratios (EDGAR) (https://edgar3.computational.bio.uni-giessen.de/cgi-bin/edgar_login.cgi?cookie_test=1) webtool. Within this analytical framework, the strain SD197 was designated as the reference for comparative assessment alongside other strains of *S*. *dysenteriae*. The core genomes, characterized by their ubiquitous presence among all examined strains, were ascertained based on shared genomic content [12].

### Identification of non-homologous and essential proteome

To eliminate core genomes that lack homology with the host organism, we utilized the Protein Basic Local Alignment Search Tool (BLASTp) (https://blast.ncbi.nlm.nih.gov/Blast.cgi?PROGRAM=blastp&PAGE_TYPE=BlastSearch&LINK_LOC=blasthome). The core genomes that did not exhibit homology with the host were then subjected to a subtractive proteomics approach aimed at identifying proteins that are non-homologous to human proteins. Subsequently, those proteins which showed no homology with human proteins were further filtered based on their essentiality using BLASTp in database of essential genes (DEG) (http://origin.tubic.org/deg/public/index.php/blast/bacteria) [13].

### Potential virulent target identification

To pinpoint potential therapeutic targets, we employed a sub-cellular localization tool known as CELLO (http://cello.life.nctu.edu.tw/) [14]. Subsequently, the proteins identified through CELLO were subjected to further filtration based on their virulence properties, utilizing Island Viewer 4. (https://www.pathogenomics.sfu.ca/islandviewer/) [15].

### Homology modelling, catalytic pocket detection and structural analysis

The prioritized potential therapeutic drug target proteins were modelled by SWISS MODELL (https://swissmodel.expasy.org/). SWISS-MODEL (http://swissmodel.expasy.org) is an online server designed for automated comparative modelling of three-dimensional (3D) protein structures. By utilizing this web tool, users can obtain the 3D structures of their target proteins [16]. DoGSiteScorer was utilized to evaluate the druggability of the obtained 3D structures. This tool is capable of detecting potential binding pockets and sub-pockets within a protein of interest. It predicts catalytic pockets and assigns a druggability score ranging from 0 to 1. A score closer to 1 indicates a highly druggable protein pocket, which is considered the most favourable for targeting with potential therapeutics [17]. Furthermore, the protein structure was validated by GalaxyRefine, Ramachandran plot and ERRAT score [13].

### Molecular docking analysis

The protein-ligand docking is to anticipate the position and orientation of a ligand within the binding pocket of a certain receptor. The docking of proteins to their ligands is a common technique used in contemporary drug development. The Molecular Operating Environment (MOE) (http://www.chemcomp.com/) software latest version MOE 2022.02 was utilised to ascertain the ligand interaction with receptor and to visualise docking of ligand molecules against protein active sites. The Chemical Computing Group has developed MOE with the purpose of providing comprehensive support for various scientific fields, including cheminformatics, molecular modelling, bioinformatics, virtual screening, and structure-based drug design [18].

Protein-ligand docking was carried out utilizing MOE with default parameters, involving the preparation and minimization of both the proteins and ligand compounds. The software provided docking scores (S), the count of interactions, and Root Mean Square Deviation (RMSD) measurements for each ligand. These criteria were employed to identify the most promising compounds, specifically those with the highest S score and interactions.

### Ligands preparation

A total of 105 ligands were retrieved from the literature. These ligands had previously been employed as antimicrobial agents in various studies but had not been tested against *S*. *dysenteriae* (Ambrogi et al. 1970; Ibis et al. 2013; Sanchez-Calvo et al. 2016; Park et al. 2006; Shan et al. 2008) [13]. The energy minimization of ligand molecules was conducted through the MOE tools Energy minimization algorithm. The energy minimization process employed the following parameters: gradient set to 0.05, force field applied was MMFF94X, and chiral constraint was set to current geometry. The resulting minimized molecules were then saved in the (.mdb) file format. In the subsequent step, these prepared ligands served as input files for MOE-Dock [19].

### Protein preparation

The protein molecules (Integration host factor subunit alpha and Tyrosine recombinase XerC) utilized in our study were modelled by SWISS-MODELL. The 3D protonation of the protein molecules was conducted. Subsequently, we performed energy minimization on the protein structures using the MOE tools energy minimization algorithm. This process employed the following parameters: gradient set at 0.05, force field applied was MMFF94X+Solvation, and chiral constraint was set to current geometry. The energy minimization process terminated upon achieving a root mean square gradient below 0.05. The resulting minimized structures served as templates for the docking process [20].

### Visualization of docked complexes

The selected ligands were then subjected to analysis using PyMOL, an open-source molecular visualization system. PyMOL, which can produce images and films of the highest quality, successfully represents macromolecules in a variety of 3D representations, such as ribbons, dots, cartoons, surfaces, sticks, and lines [18].

## Results

### Genome sequence and pan-genome retrieval

Pan-genomic EDGAR webtool was utilized for comparative analysis with other *S*. *dysenteriae* strains, we selected the strain SD197 as the reference strain. A total Pan-genomes were 3769 whereas, 2618 core genomes which were shared among all strains. These core genes were used for further downstream analysis.

### Pan-genomic identification of non-homologous, essential proteome and potential targets

Total core genomes undergo subtractive analysis on BLASTp to retrieve non-host homologous proteins, there were 1603 non-host homologous proteins that are further characterized on the basis of their essentiality that resulted in 195 essential proteins that were filtered out by BLASTp at database of essential gene (DEG). The essential proteins were analysed further for the identification of potential drug targets. By utilizing the CELLO database, we conducted an analysis of subcellular localization, allowing us to distinguish proteins based on their precise locations within the bacterial cell. Our findings revealed a total of 137 cytoplasmic proteins, 21 periplasmic proteins, 34 inner membrane proteins, and 2 extracellular proteins.

### Potential virulent target identification

The virulence factor of each protein was assessed using IslandViewer 4, leading to the identification of two novel cytoplasmic virulent proteins. In the selection of drug targets, cytoplasmic proteins are regarded as highly favourable candidates. Therefore, these 2 targets have a high potential for being druggable (Table 1). Proteins that are both functionally characterized as virulent and essential play a crucial role in identifying potential therapeutic targets.

**Table 1:** Functional properties of prioritized drug targets.

Target proteins	UniProt ID	Amino acid	Virulence	Gene	Biological function	Molecular function
**Integration host factor subunit alpha**	Q32FI7	99	Yes	ihfA	Regulation of DNA template. Transcription. Regulation of translation.	Structural constituent of chromatin.
**Tyrosine recombinase XerC**	Q329Y7	298	Yes	Xerc	Cell division. Chromosome segregation.	DNA binding. Tyroxsine based site specific recombinase activity

### Structural analysis of prioritized drug targets

**Fig 1:**
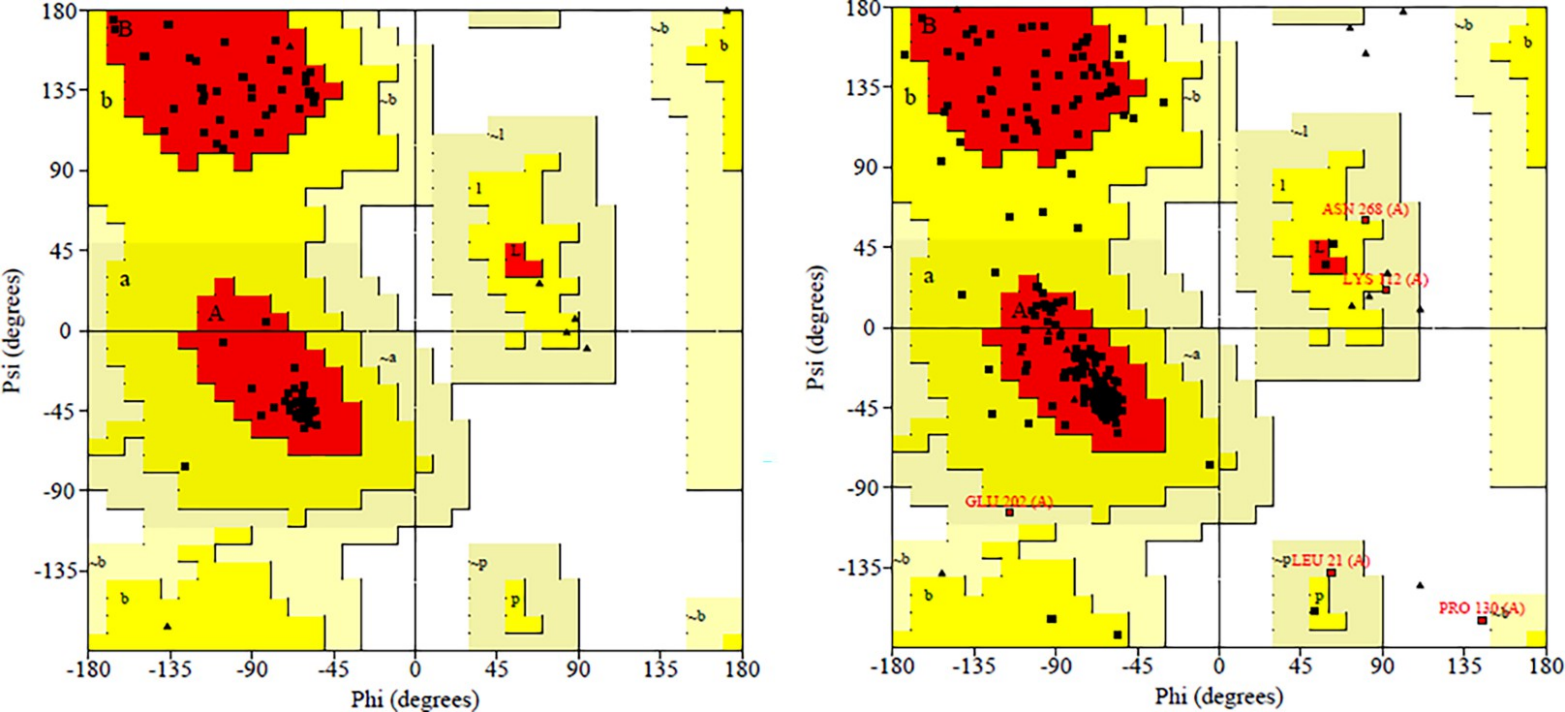
Conformational analysis via ramachandran plots: Prioritized drug target proteins. (A) Integration Host Factor Subunit Protein and (B) Tyrosine Recombinase XerC.

The potential therapeutic drug target proteins were modelled on SWISS-MODELL that were prioritized namely Integration host factor subunit alpha and Tyrosine recombinase XerC and were validated by GalaxyRefine, ERRAT and Ramachandran plot. Similarly, Ramachandran plot of Integration host factor subunit showing in red colour indicating (Fig 1A**)** residues in most favoured region is 98.8% and allowed regions is 1.2%. Ramachandran Plot of Tyrosine recombinase XerC showing in red colour indicating residues in most favoured region is 90.2% and allowed regions is 8.3% (Fig 1B). This high score indicates that the protein structure possesses a high-resolution representation.

**Fig 2:**
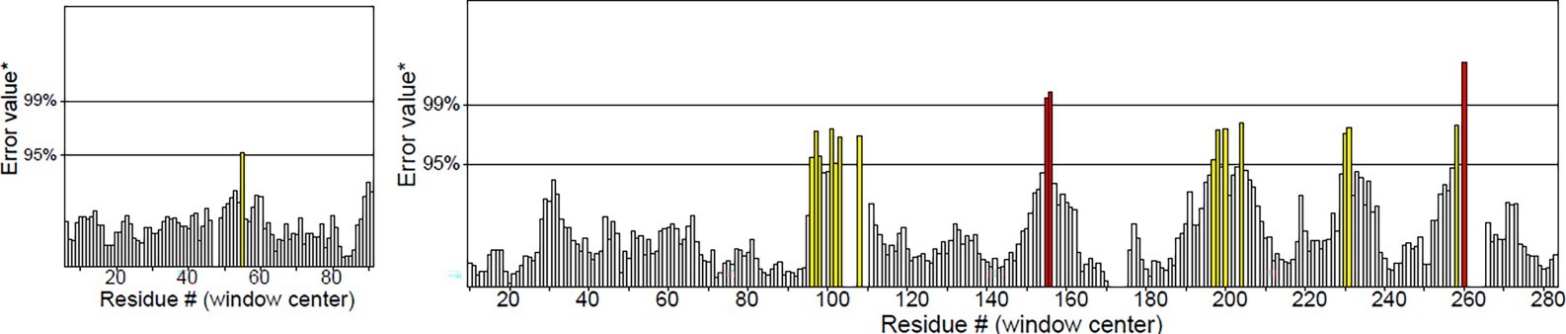
Quality assessment of protein structures using ERRAT tool. (A) Integration Host Factor Subunit: Score of 98.8%, (B) Tyrosine Recombinase XerC: Score of 93.3%".

The overall quality factor of 3D structure was predicted by ERRAT. ERRAT score of Integration host factor subunit was 98.8% and Tyrosine recombinase XerC was 93.3% (Fig 2A and 2B**).** On the labelled axis representing "error," there are two lines that indicate the extent of certainty in excluding regions where the error values exceed the specified threshold. Residues with error values surpassing 99% were identified and marked in red, while areas with error values greater than 95% are indicated in yellow.

### Docking analysis of prioritized drug targets

**Fig 3:**
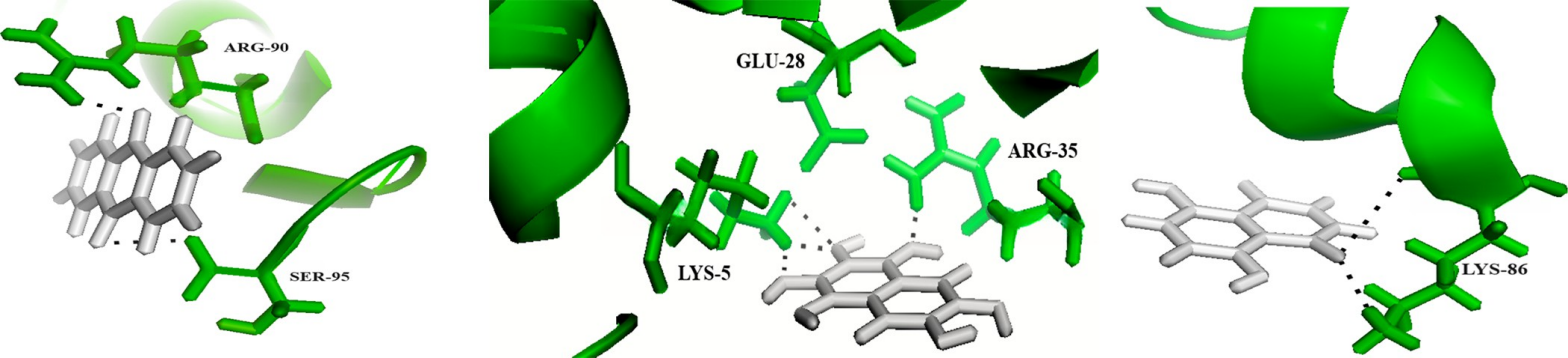


A total of 105 plant-derived compounds were subjected to protein docking in this study (S1 File). Among these, four top-ranking ligands were carefully chosen, demonstrating a robust binding affinity with specific drug targets. The interaction between protein and compound was evaluated using several scores such as RMSD score, docking score.

(Fig 3A) The interaction between anthraquinone (shown in white) and Integration Host Factor Subunit Alpha is illustrated in the figure. The residues involved in the interaction are highlighted in purple, while the bonds formed with the ligand are represented by black dotted lines. The RMSD score, which measures the deviation between the docked and experimental structures, was determined to be 0.4718 Å. The minimized energy score, indicating the stability of the interaction, was -19.903 kcal/mol. Additionally, the docking score, which reflects the quality of the docking pose, was determined to be -6.5728.

Another interaction involving the Integration host factor subunit alpha, this time with 5-hydroxy-1,4-naphthalenedione, was also analysed.

**(**Fig 3B) The diagram depicts the interaction between 5-hydroxy-1,4-naphthalenedione (displayed in white) and Integration Host Factor Subunit Alpha. Residues engaged in this interaction are highlighted in purple, and bonds with the ligand are denoted by black dotted lines. The RMSD score for this interaction was found to be 0.2796 Å. The minimized energy score was -16.914 kcal/mol, suggesting a favourable energy state. The docking score was -10.3786, indicating a strong docking pose.

Finally, the interaction between the Integration host factor subunit alpha and 5,8-dihydroxynaphthalene-1,4-dione (Fig 3C). The figure illustrates the interaction between 5,8-dihydroxynaphthalene-1,4-dione and Integration Host Factor Subunit Alpha. Residues participating in this interaction are emphasized in purple, while bonds to the ligand are depicted as black dotted lines. Exhibited an RMSD score of 0.6007 Å. The minimized energy score was -19.806 kcal/mol, implying a stable interaction. The docking score was -8.4463, suggesting a reasonably good docking pose. All the interactions, interacting residues, RMSD values and docking score is shown in Table 2.

**Table 2:** Interaction profiles of anthraquinone, 5-Hydroxy-1,4-Naphthalenedione, and 5,8-Dihydroxynaphthalene-1,4-Dione with Integration host factor subunit alpha: Protein-ligand interaction analysis.

Compound	Protein	Number of interactions	Interacting residues	Minimized energy	Docked score	RMSD
**Anthraquinone**	Integration host factor subunit alpha	2	ARG-90, SER-95	-19.903 kcal/mol	-6.5728	0.4718
**5-hydroxy-1,4-naphthalenedione**	Integration host factor subunit alpha	3	GLU-28, LYS-5, ARG-35	-16.914 kcal/mol	-10.3786	0.2796
**5,8-dihydroxynaphthalene-1,4-dione**	Integration host factor subunit alpha	1	LYS-86	-19.806 kcal/mol	-8.4463	0.6007

**Fig 4:**
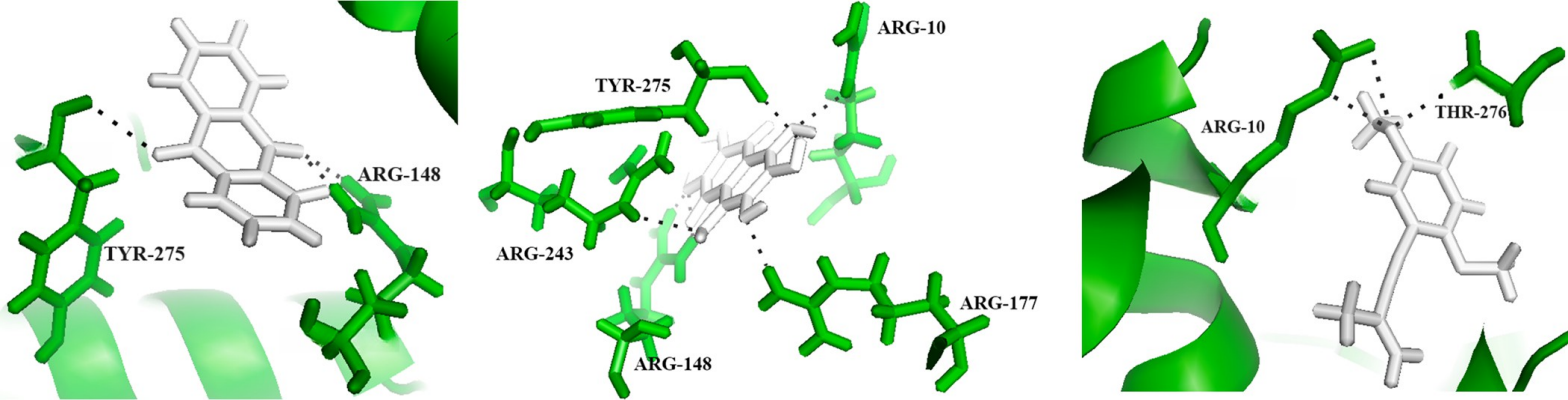


The interaction between anthraquinone and Tyrosine recombinase XerC is shown in Table 3, and the results indicate a RMSD score of 0.7835 Å. The minimized energy for this interaction was determined to be -5.526 kcal/mol, suggesting a relatively stable state. The docked score was -7.8689 (Fig 4A). Showcases the interaction between anthraquinone (shown in white) and Tyrosine recombinase XerC. The residues engaged in this interaction are highlighted in purple, forming bonds with the ligand, as illustrated by black dotted lines.

**Table 3:** Table presents the interaction profiles of Anthraquinone, 5-hydroxy-1,4-naphthalenedione, and 1,4-dimethoxy-2-(2-methylbut-3-en-1-ynyl) benzene with Tyrosine recombinase XerC. Each compound’s interactions with Tyrosine recombinase XerC are summarized, providing insights into their binding affinities and molecular interactions.

Compound	Protein	Number of interactions	Interacting residues	Minimized energy	Docked score	RMSD
**Anthraquinone**	Tyrosine recombinase XerC	2	ARG-148, TYR-275	-5.526 kcal/mol	-7.8689	0.7835
**5-hydroxy-1,4-naphthalenedione**	Tyrosine recombinase XerC	5	ARG-10, ARG-177, ARG-148, ARG-243, TYR-275	-23.030 kcal/mol	-15.2267	0.5119
**1,4-dimethoxy-2-(2-methylbut-3-en-1-ynyl) benzene**	Tyrosine recombinase XerC	2	ARG-10, THR-276	-18.968 kcal/mol	-10.9519	0.3504

Similarly, the interaction of 5-hydroxy-1,4-naphthalenedione with Tyrosine recombinase XerC was evaluated (Fig 4B). Illustrates the interaction between 5-hydroxy-1,4-naphthalenedione (depicted in white) and Tyrosine recombinase XerC. The residues participating in this interaction are highlighted in purple, indicating their involvement in forming bonds with the ligand, as depicted by black dotted lines.

The RMSD score for this interaction was found to be 0.5119 Å. The minimized energy score was -23.030 kcal/mol. The docked score was -15.2267.

Lastly, the interaction between 1,4-dimethoxy-2-(2-methylbut-3-en-1-ynyl) benzene and Tyrosine recombinase XerC exhibited an RMSD score of 0.3504 Å. The minimized energy score was -18.968 kcal/mol, implying a relatively stable interaction. The docked score was -10.9519 (Fig 4C**)**. Demonstrates the interaction between 1,4-dimethoxy-2-(2-methylbut-3-en-1-ynyl) benzene (presented in white) and Tyrosine recombinase XerC. Residues engaged in this interaction are highlighted in purple, indicating their involvement in forming bonds with the ligand, as depicted by black dotted lines. The interactions and RMSD values exhibited by 5-hydroxy-1,4-naphthalenedione against both proteins were highly satisfactory (S1 File).

## Discussion

It has been documented that approximately 200,000 deaths annually annual deaths due to diarrhoea are associated with shigellosis, presenting a significant global public health concern [21]. *Shigella* species are diarrheal pathogens closely linked to *Escherichia coli*. They owe their name to Kiyoshi Shiga, who, in 1898, identified the most virulent member, *S*. *dysenteriae*, as the causative agent of bacillary dysentery, also recognized as shigellosis. *Shigella* are typically transmitted via the fecal-oral route or through the consumption of contaminated food and water. While most cases of *Shigella spp*. result in a self-constraining disease, which can be managed effectively through oral rehydration or antibiotic treatment, but there has been a concerning rise in shigellosis instances caused by antibiotic-resistant strains of *Shigella*, a major health concerning issue [22]. Advances in bioinformatics have enabled the prediction of potential new therapeutic options with a high degree of accuracy [23].

In our present study, we employed a subtractive and comparative genomic analysis approach to identify a total of 2618 core proteins, these proteins are uniquely found in *S*. *dysenteriae*. Utilizing comparative genomics and subtractive genomics methodologies, novel vaccine and drug targets have been identified. presenting pathway for addressing this serious public health issue. Specifically, two non-host homologous proteins have been identified as potential drug targets. Through molecular docking analysis, Anthraquinone, 5-hydroxy-1,4-naphthalenedione, and 1,4-dimethoxy-2-(2-methylbut-3-en-1-ynyl) benzene have emerged as compounds demonstrating optimal interactions with the identified drug targets, thereby showcasing considerable promise for further exploration and potential therapeutic development. Studies have documented the significant efficacy of Anthraquinones against Gram-negative bacteria, showcasing their pronounced antimicrobial activity. The documented evidence supports the synergistic effect of anthraquinones with other antibiotics, leading to a reduced Minimum Inhibitory Concentration (MIC) [24]. Naphthoquinones exhibit multiple properties, demonstrating remarkable potential in inhibiting bacteria, fungi, parasites, and cancer cells. Additionally, Gram-positive pathogens are particularly susceptible to the effects of naphthoquinones. Chansukh K et al., showed a significant inhibition of *Shigella*. *Spp* by quinone derivatives [25]. Extracted from Onosma, a member of the Boraginaceae family [26], 5,8-dihydroxynaphthalene-1,4-dione is acknowledged as an eco-friendly reagent possessing diverse properties including antibacterial, antimalarial, anticancer, and herbicidal activities. Research has indicated its ability to inhibit both gram-negative and gram-positive bacteria. This compound exhibited notable antibacterial efficacy against *Salmonella typhi* and *S*. *dysenteriae*, with a noteworthy activity observed against *S*. *dysenteriae* (21 mm) [27]. Finally, in future research endeavours aimed at developing drugs to combat Shigellosis disease, these compounds could be deemed worthy of consideration for antimicrobial therapy.

## Conclusions

Due to the emergence of resistance genes in *S*. *dysenteriae*, the effectiveness of current drugs against this bacterium is diminishing. To address this issue, we conducted a study aimed at identifying potent drug targets and exploring alternative approaches using plant-derived compounds to suppress the disease. The study employed *in-silico* methodology, which allowed for the identification of potential drug targets through computational analysis. In our study, we conducted a pan-genomic analysis involving 27 strains of *S*. *dysenteriae*, leading to the identification of 2618 core proteins shared among these strains. Through subtractive genomic analysis and determination of essential genes, we were able to narrow down the potential targets by focusing on specific hits. These selected targets play crucial roles in the survival and virulence of the bacteria, providing valuable insights for selecting the most promising therapeutic targets. To further explore potential treatments, we performed molecular docking of the identified targets with plant-derived compounds obtained from the literature. Based on the interaction of residues and docking scores, we filtered out the selection to four compounds that showed the most favourable interactions with the targets. To validate these findings, experimental studies are essential to validate the results we obtained *in silico* and to further evaluate the efficacy of the selected compounds.

## Supporting information

S1 File Supporting compounds, docked complex. (ZIP)
